# Means of Arresting Hæmorrhage after Excision of Tonsils

**Published:** 1846-05-01

**Authors:** 


*Means of arresting Hemorrhage after excision of Tonsils.*—It is well known that occasionally after the operation for excision of the tonsils, hemorrhage is troublesome and has even proved fatal. Prof. Hamilton stated at a recent meeting of the Buffalo Medical Association, that he had always found cold applications to the neck externally, immediately effectual in cases of this accident. He prefers, when circumstances will permit, snow enveloped in a neck cloth. Astringent gargles he thinks often do as much harm as good by disturbing the formation of coagula.

